# TL-DETR: Efficient transmission line defect detection for edge deployment

**DOI:** 10.1371/journal.pone.0351470

**Published:** 2026-06-11

**Authors:** Yong Zhang, Runming Zhao

**Affiliations:** School of Computer and Artificial Intelligence, Beijing Technology and Business University, Beijing, China; University of South China, CHINA

## Abstract

Visual inspection is critical for power system maintenance, yet deploying high-performance detection models on resource-constrained edge devices remains challenging due to complex background interference, extreme defect scale variations, and high computational overhead. This paper presents TL-DETR, a specialized detection framework that integrates multi-scale feature enhancement and dynamic sparse attention to achieve accurate and efficient transmission line defect detection for edge deployment. First, a ResNet-50-TL backbone network incorporating a multi-scale feature enhancement module is designed to preserve fine-grained features. Subsequently, the neck network integrates Attention-based Intra-scale Bi-level Routing and a channel shuffle mechanism to precisely focus on critical defects and reduce parameter count. Furthermore, a multi-scale attention mechanism is introduced to accomplish pixel-level recalibration through cross-spatial learning. Experiments on the CableInspect-ADs dataset demonstrate that the precision and mAP50 of TL-DETR reach 91.4% and 86.0%, respectively, representing improvements of 3.2% and 2.9% over the baseline RT-DETR. These results indicate that the model effectively balances accuracy and computational efficiency, demonstrating theoretical viability for practical edge deployment. Generalization experiments confirm that the model exhibits excellent generalization capabilities for detecting insulators, vibration dampers, and bolts, aligning closely with the engineering requirements for precise perception of minute defects.

## Introduction

Modern power systems, which serve as the energy arteries supporting societal operations, are experiencing a synergistic advancement of scale expansion and technological innovation [1]. With the construction of the global energy internet and the extensive integration of renewable energy, the scale of power transmission infrastructure has reached an unprecedented level. According to the latest report from the International Energy Agency, to achieve the energy transition and climate goals of various countries, more than 80 million kilometers of new or refurbished transmission and distribution lines will be needed globally by 2040 [2]. As the core channels for power delivery, the safety and stability of transmission lines are directly related to the reliable operation of the entire power grid, necessitating regular and meticulous inspections [3–5]. However, the explosive growth in the scale of the power grid has brought severe challenges to traditional operation and maintenance models; the per capita inspection mileage for personnel is rising year by year, and the traditional manual inspection mode is inadequate to address the growing maintenance demands [6]. Therefore, seeking efficient and safe automated inspection technologies has become an urgent requirement of the industry.

In recent years, computer vision-based automated defect detection technology has evolved from traditional machine learning that relies on manually designed features (such as Haar features [7] and Histogram of Oriented Gradients [8]) to deep learning-driven approaches. Convolutional Neural Networks (CNNs) represented by the R-CNN series [9–12], YOLO [13], and SSD [14] have significantly improved detection accuracy and speed. Meanwhile, with the introduction of the Transformer [15], DETR [16], and its derivative model RT-DETR (Real-Time Detection Transformer) [17], these models achieved further breakthroughs in vision tasks, thanks to their “set prediction” and end-to-end advantages. With the application of deep learning in power line inspection, existing research has made significant progress, and its technical focus is shifting from “centralized high-precision computing in the cloud” to “high-efficiency collaboration at the edge”. In the pursuit of accuracy, many solutions relied heavily on “centralized cloud processing” to support complex network designs. For example, Aohua Li et al.[18] designed a cascaded framework combining YOLOv5s and U-Net, which effectively improved the segmentation performance of defect regions. To address dense small-object detection in complex backgrounds, both TLI-DETR and PLD-DETR [19,20] enhanced their perception of key transmission line components by introducing attention mechanisms and feature fusion modules. For specific transmission line fittings, Wenxia Bao et al.[21] and Zhang Ke et al.[22] proposed PMA-YOLO and a knowledge-reasoning-based PA-DETR, respectively, which effectively addressed the discrimination challenges posed by small vibration damper targets and visually indistinguishable bolts. However, when facing massive concurrent uploads of high-definition images, these computation-intensive models are highly prone to network congestion, and their data transmission latency severely hinders real-time feedback on faults. To break through this timeliness bottleneck, the “cloud-edge-device collaborative” mode, which pushes computing power to the edge, has become an inevitable trend in automated inspection. Many scholars have begun seeking a balance between speed and accuracy under limited edge-computing power constraints. For instance, to address the challenges posed by complex backgrounds and occlusions, Jinyu Wang et al.[23] used statistical prior information to construct an enhanced DETR framework that improves the speed of small-object detection. Pei Dongfeng et al.[24] proposed the RT-DETR-MBD model by constructing a lightweight backbone, achieving highly efficient edge detection while significantly reducing parameter count. Furthermore, for lightweight identification of specific defects, Jinxing Niu et al.[25] and Zhilong Yu et al.[26] proposed the SL-YOLOv8 and DFCG_YOLOv5 algorithms, respectively, incorporating filtering and noise reduction, achieving dual improvements in accuracy and speed on engineering data.

With the widespread adoption of deep learning in industry, researchers have begun to explore more generalizable and interpretable advanced condition-monitoring and diagnosis paradigms. For example, in the field of intelligent diagnosis of complex industrial equipment, multimodal data fusion technology has made significant progress. You et al. constructed a heterogeneous fusion model based on liquid-impulse neural networks to enhance temporal feature extraction [27], and achieved intelligent fusion and interpretability quantification of acoustic-vibration signals through an attention mechanism [28]. Furthermore, to overcome the difficulty of scarce real fault samples, physics-informed neural networks (PINN) have been introduced into data-driven networks, enabling high-precision diagnosis under zero-fault sample conditions [29]; further, a multi-edge physical information digital twin framework demonstrated excellent diagnostic performance through the dynamic interaction of virtual and real signals, even without real fault samples and under strong noise interference [30]. These cutting-edge studies provide high-precision, highly robust solutions for the intelligent operation and maintenance of critical industrial equipment, and also offer valuable theoretical references for the intelligent upgrade of power systems.

However, constrained by the single visual perception of UAVs and the limitations of edge computing, the aforementioned advanced models that rely on multi-sensor data or complex simulations are difficult to implement, and current inspection tasks still heavily rely on pure-vision lightweight detection models. Nevertheless, existing models still face three major limitations in practical applications: firstly, there is a natural contradiction between computing power and perception, and lightweight design is usually achieved at the expense of the depth and breadth of feature extraction; secondly, fine-grained features are highly susceptible to loss, and when confronting weak defects highly integrated with complex backgrounds, such as broken or spaced strands, the model’s perceptual capability decreases sharply, making it prone to missed or false detections; finally, public datasets are scarce and defect types are simplistic, with existing research mostly restricted to single components and binary classifications of “presence or absence of defects,” lacking the support of a refined, full-chain dataset and a comprehensive detection system.

To address the above issues, this paper proposes TL-DETR (Transmission-Line Detection Transformer), a high-accuracy transmission-line defect-detection model designed for edge devices. Unlike methods that solely pursue extreme lightweighting, this study aims to maximize the model’s perception capability for fine-grained defects while maintaining an acceptable level of edge computing power. The main contributions of this paper are as follows:

1. Dataset Enhancement and Construction: Based on real natural environment backgrounds, the CableInspect-ADs overhead line dataset was expanded and constructed, and the Transmission-Line-Fittings dataset covering insulators, bolts, and vibration dampers was collected and built, forming a complete chain of transmission line datasets.
2. Multi-Scale Feature Enhancement: The ResNet-50 backbone network is enhanced by introducing a Multi-Scale Feature Enhancement (MSFE) module. Through parallel multi-scale pathways based on Depthwise Separable Convolution (DWConv), the effective receptive field is expanded with extremely low computational overhead, preserving the geometric details of minute defects from the source.
3. Hybrid Attention Neck Optimization: First, an Attention-based Intra-scale Bi-level Routing (AIBR), adapted from BiFormer, is utilized to filter background noise and focus on defects through dynamic sparse computation. Second, a ShuffleC3 module is integrated to significantly reduce parameter redundancy via a channel-shuffling mechanism. Finally, an Efficient Multi-Scale Attention (EMA) is introduced to perform feature recalibration via cross-spatial learning. The integration of these three components enables high-accuracy minute-object capture at low computational cost.
4. Experimental Validation: Experiments on the aforementioned self-built datasets demonstrate that TL-DETR outperforms existing mainstream methods in key metrics such as precision, recall, and mAP50, achieving an excellent balance between detection accuracy and inference speed at the edge.

### Related work

#### Baseline model RT-DETR.

The core design philosophy of RT-DETR (Real-Time Detection Transformer) [17] is to address the high computational complexity and slow inference speed inherent in traditional DETR models. Consequently, it proposes an “efficient hybrid encoder” architecture that applies the Transformer’s attention mechanism exclusively to high-level features while using convolutional networks for multi-scale fusion.

**Fig 1:**
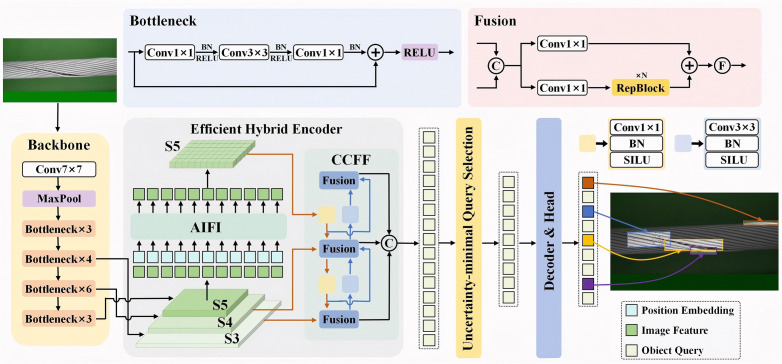
Workflow of the RT-DETR model.

The flowchart of the RT-DETR model is shown in Fig 1, and its workflow can be divided into three parts.

The first part is the backbone network, employing ResNet-50 to extract image features. It outputs three feature maps of different scales, denoted as S3, S4, and S5. S3 has a higher resolution (1/8 of the original image) and contains abundant geometric details, such as edges and textures, but weaker semantic information; S4 has a medium resolution (1/16); and S5 has the lowest resolution (1/32) but contains the richest high-level semantic information, despite relatively blurred spatial positioning.

The second part is the Efficient Hybrid Encoder, representing the core innovation of RT-DETR. It splits the encoder into AIFI (Attention-based Intra-scale Feature Interaction) and CCFF (Cross-scale Feature-fusion) modules. Specifically, the AIFI module processes only the highest-level S5 feature map using the Transformer’s self-attention to capture global context, effectively avoiding the massive computational overhead of calculating attention across all scales. The CCFF module adopts a convolution-based cross-scale fusion strategy (similar to FPN/PANet), bi-directionally fusing high-level semantic features from AIFI with low- and medium-level detail features (S3, S4) to generate high-quality representations that capture both global semantics and local details.

The third part is the Transformer Decoder & Head, responsible for final object prediction. RT-DETR introduces a “query selection” mechanism, abandoning static, zero-initialized queries from the traditional DETR framework. Instead, it directly filters the top-K features with the highest confidence scores from the encoder’s output to serve as initial object queries, performing an efficient “pre-screening.” Subsequently, these queries are fed into the multi-layer Transformer decoder, which continuously interacts with and updates image features via the cross-attention mechanism. Finally, the prediction head directly outputs object categories and bounding boxes without Non-Maximum Suppression (NMS) post-processing, achieving true end-to-end, real-time detection.

### Attention mechanism

In the specific scenario of transmission line inspection, images are typically characterized by complex backgrounds and minute defect scales. This “low signal-to-noise ratio” characteristic makes it difficult for traditional convolutional networks to accurately focus on minute defects while suppressing the background. To address this issue, attention mechanisms have been widely introduced, aiming to simulate the human visual system’s ability to focus on salient regions, thereby enhancing target features and suppressing irrelevant backgrounds through dynamic weight allocation.

The attention mechanism has become a key technology for improving the feature extraction efficiency of deep learning models by simulating the human visual system’s ability to focus on salient regions. In early convolutional neural networks, SE-Net (Squeeze-and-Excitation Networks) [31] significantly improved model performance by reweighting channel attention. Subsequently, CBAM (Convolutional Block Attention Module) [32] further integrated spatial and channel dimensions, achieving finer feature calibration. Shuai Hao et al.[33] introduced the CBAM attention mechanism into a transmission line inspection model, effectively improving its accuracy by embedding a spatial- and channel-adaptive feature enhancement module into the backbone network.

Meanwhile, with the introduction of the Transformer architecture, the Self-Attention mechanism has achieved breakthroughs in object detection tasks by virtue of its powerful global context modeling [15]. However, traditional Global Self-Attention faces a massive computational burden, as its computational complexity grows quadratically with the length of the input feature sequence, and it is highly susceptible to background noise interference, which is particularly fatal in the complex backgrounds of transmission lines [34]. To this end, researchers have developed various lightweight vision models oriented towards edge devices. For example, MobileViT [35] integrates the local inductive bias of convolutions with the global modeling capacity of Transformers; EdgeFormer [36] significantly reduces on-device resource consumption through a parameter-efficient design; EfficientFormer [37] elevates Transformer inference speed to the level of traditional CNNs by relying on a latency-driven slimming strategy; furthermore, the classic Swin Transformer [38] introduces a local self-attention mechanism based on shifted windows, successfully reducing the quadratic computational complexity of global attention to linear, thereby providing an efficient paradigm for lightweight design.

Beyond architectural lightweight explorations, recent studies have also focused on targeted attention optimizations in defect detection to address the challenges posed by complex transmission line backgrounds and the high susceptibility of small object features to loss. For instance, to tackle the problem of small object feature loss in complex backgrounds, the aforementioned TLI-DETR model [19] significantly enhances the model’s perceptual capability for subtle faults by introducing a channel-spatial fusion cross-attention mechanism; the PLD-DETR model [20] further introduces a Local Cross Attention module, which effectively suppresses redundant noise interference in the backgrounds of transmission line insulator strings and towers by focusing on local feature interactions. Aiming at the missed detection of small objects caused by complex backgrounds and object occlusion in UAV inspection images, Tan et al. [39] derived multi-scale attention feature maps based on an ultra-lightweight subspace attention mechanism (ULSAM) and combined them with Soft-NMS to significantly reduce the missed detection rate. Meanwhile, facing the challenges of scarce industrial surface defect samples and variable appearances, the MaMiNet model proposed by Luo et al. [40] introduced a Memory Attention feature enhancement module, which effectively improves the network’s discriminative feature representation capability for various defect regions by capturing attention information across samples. Furthermore, the MAP-YOLOv8 model proposed by Xu et al. [41] employs a Mixed Pooling Channel Attention mechanism that effectively integrates global and local contextual information while preserving key texture details by aggregating global average pooling and maximum pooling features in parallel, thereby further improving recognition accuracy.

To further retain key features while reducing computational complexity, researchers have proposed various sparse attention paradigms. For example, SparseViT [42] introduces a dynamic pruning mechanism based on importance scoring, which highly concentrates computational resources on foreground objects by progressively eliminating low-information background tokens in deep networks. In other evolutions of the DETR series models, the optimization of computational efficiency and accuracy is also a core direction: Deformable DETR [34] proposes a deformable sampling mechanism that effectively alleviates computational pressure and accelerates model convergence by focusing only on a small set of key sampling points near reference points; Dynamic DETR [43] achieves adaptive adjustment across different scales, spatial locations, and feature attributes by introducing multi-dimensional dynamic attention, which further enhances perceptual accuracy. In addition, the Bi-Level Routing Attention proposed by BiFormer [44] dynamically constructs routing graphs to compute attention only among semantically relevant regions. This “dynamic sparse” strategy not only drastically reduces GPU memory overhead but also better aligns with the edge deployment requirements of transmission line inspections.

## Methods

### Improved TL-DETR

**Fig 2:**
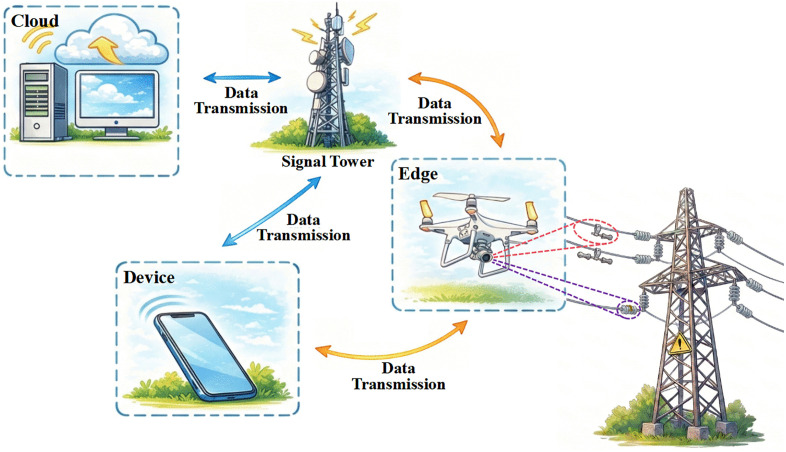
Schematic diagram of the cloud-edge-device collaborative architecture.

In the actual inspection process of transmission lines, the ideal approach is a “cloud-edge-device collaborative architecture,” as shown in Fig 2, where the object detection algorithm is deployed on edge devices to process data, thereby improving inspection efficiency. To meet the lightweight requirements of edge deployment and to resolve the issues of feature extraction redundancy and insufficient perception of small objects inherent in the original RT-DETR model when processing fine-grained industrial defects, this paper proposes an improved model—TL-DETR. While retaining the real-time advantages of the baseline RT-DETR-ResNet50 architecture, this model focuses on the targeted adaptation and optimization of the backbone and neck networks. Its core improvements comprise the following three aspects:

First, the backbone network is improved. To address the issue where the traditional ResNet-50 easily loses minute object details during continuous downsampling and the receptive field of a single 3 × 3 convolution is limited, a ResNet-50-TL backbone network incorporating an MSFE (Multi-Scale Feature Enhancement) module is constructed. By replacing the last residual block of each stage, the MSFE introduces multi-path parallel branches to expand the effective receptive field. Meanwhile, to prevent a surge in computational overhead caused by large-scale feature extraction, this module comprehensively adopts Depthwise Separable Convolutions (DWConv), thereby strengthening the capture of minute defects from the source at an extremely low parameter cost and ultimately achieving a balance between high perception performance and edge lightweighting.

Second, a dynamic sparse and lightweight neck is adapted. In the Hybrid Encoder stage, the AIBR module is initially introduced to replace the original AIFI module, aiming to reconstruct the high-level feature interaction mechanism. Through dynamic sparse computation, it automatically filters redundant noise in the power transmission background, achieving precise focusing on critical defect regions. Simultaneously, a ShuffleC3 module based on a channel shuffle mechanism is designed to replace the original RepC3. This significantly reduces the model’s parameter count and computational complexity while preserving the flow of feature information, making it well-suited for edge deployment.

**Fig 3:**
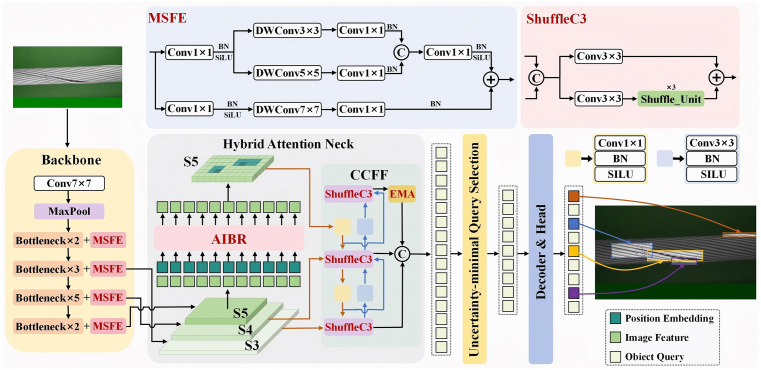
Workflow of the TL-DETR model.

Third, global feature recalibration is conducted. The EMA module is introduced at the end of the feature fusion process. By leveraging its unique Cross-Spatial Learning mechanism to aggregate global contextual information, it performs pixel-level recalibration and purification of the output features, further resolving the issue of insufficient localization precision for minute defects. Workflow of the TL-DETR model as shown in Fig 3.

### Improvement of the ResNet-50-TL backbone network

**Fig 4:**
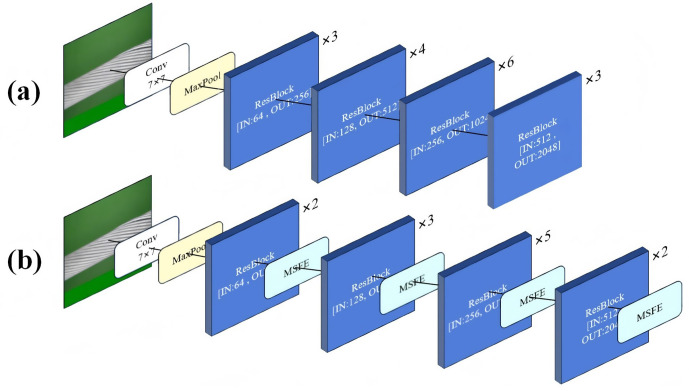
Structure of the backbone network: (a) ResNet-50; (b) ResNet-50-TL.

Given the minute scales and complex textures of defect targets (e.g., broken strands and wear) in transmission line inspection scenarios, the traditional ResNet-50 backbone network suffers from dual limitations: high-frequency detail loss and fixed receptive fields during continuous downsampling. To resolve this issue, this paper proposes an improved ResNet-50-TL backbone network. A comparison between ResNet-50 and ResNet-50-TL is shown in Fig 4. Its core strategy is to introduce the MSFE module at the end of Stage 2 through Stage 4 in the original network, replacing the standard residual blocks. The MSFE adopts a “split-transform-fuse” parallel topology, which essentially arranges multi-branch depthwise separable convolutions in parallel, to work synergistically to enhance the model’s representation of minute defects from the source.

**Fig 5:**
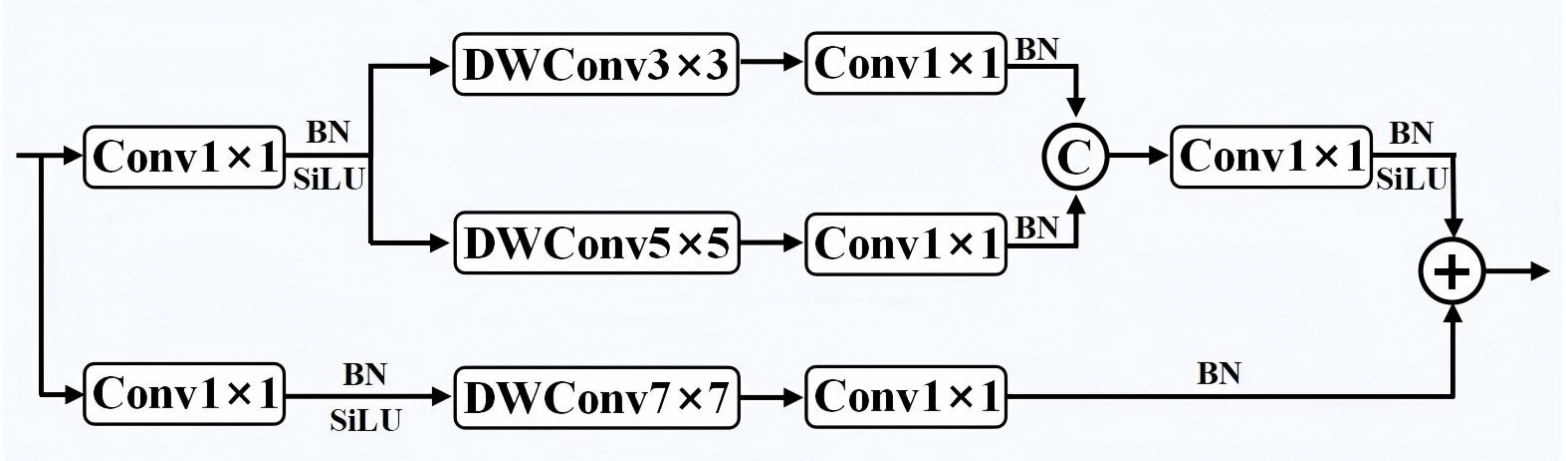
Structure of the MSFE module.

Specifically, the MSFE module splits the input feature $\text{X}\in R^{\text{C}\times \text{H}\times \text{W}}$ into two parallel perception branches. To multiply the receptive field while strictly controlling computational complexity for edge deployment adaptation, the MSFE module discards traditional large-kernel standard convolutions and introduces DWConv in a comprehensive manner. The fine-grained local perception branch aims to capture minute textures. It first performs dimensionality reduction through a 1 × 1 convolution (with SiLU activation), and then further splits into two paths, utilizing 3 × 3 and 5 × 5 depthwise separable convolutions (i.e., k × k DWConv for spatial extraction + 1 × 1 Conv for channel fusion) respectively to extract spatial features of different granularities. Finally, they are concatenated along the channel dimension and fused by a 1 × 1 convolution. The coarse-grained global perception branch utilizes a 7 × 7 depthwise separable convolution to capture the global context of elongated defects. The structure of the MSFE module is illustrated in Fig 5.

Mathematically, let ${\text{F}}_{\text{1}\times \text{1}}(\cdot )$ be the 1 × 1 standard convolution operator including batch normalization, ${\text{F}}_{\text{DWS}\_\text{k}\times \text{k}}$ be the depthwise separable convolution operator including k×k DWConv and 1 × 1 Conv, and σ be the SiLU activation function. The feature extraction processes of the two branches can be formally expressed as follows:


$$\begin{array}{c} {\text{Y}}_{\text{local}}=\sigma ({\text{F}}_{\text{1}\times \text{1}}(\text{Concat}[{\text{F}}_{\text{DW}{\text{S}}_{\text{3}\times \text{3}}}({\text{X}}^{\prime }),{\text{F}}_{\text{DW}{\text{S}}_{\text{5}\times \text{5}}}({\text{X}}^{\prime })])),\text{ where }{\text{X}}^{\prime }=\sigma ({\text{F}}_{\text{1}\times \text{1}}(\text{X})) \end{array}$$ (1)

$$\begin{array}{c} {\text{Y}}_{\text{global}}={\text{F}}_{\text{DWS}\_\text{7}\times \text{7}}(\sigma ({\text{F}}_{\text{1}\times \text{1}}(\text{X}))) \end{array}$$ (2)

where ${\text{X}}^{\prime }$ is the intermediate feature after dimensionality reduction preprocessing, and ${\text{Y}}_{\text{local}}$ and ${\text{Y}}_{\text{global}}$ represent the local and global feature responses, respectively. Ultimately, the module aggregates the information from the two branches through element-wise summation to form an output feature with multi-scale response capabilities:


$$\begin{array}{c} {\text{Y}}_{\text{out}}={\text{Y}}_{\text{local}}⊕{\text{Y}}_{\text{global}} \end{array}$$ (3)

Through this design, ResNet-50-TL successfully resolves the dilemma of parameter explosion caused by multi-scale large-kernel convolutions. By leveraging DWConv’s extremely high computational compression ratio, it constructs a parallel, synergistic feature pyramid-style spatial response mechanism without introducing computational burden. This effectively solves the problem of feature submergence caused by minute defects in deep networks, achieving high-quality feature extraction optimized for edge devices.

### Hybrid attention neck optimization

#### Dynamic sparse attention based on intra-scale bi-level routing.

**Fig 6:**
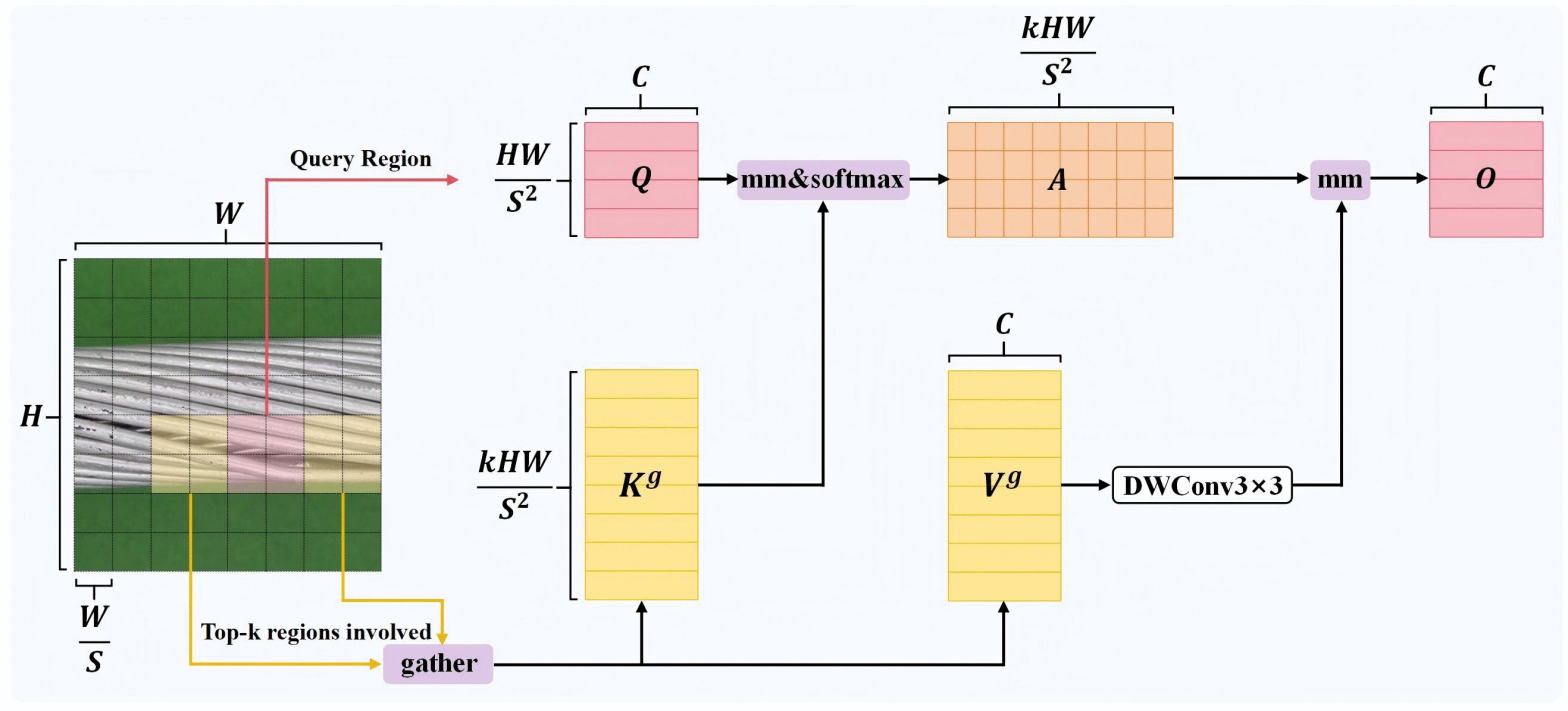
Network architecture of the Attention-based Intra-scale Bi-level Routing.

In transmission line inspection images, key targets such as overhead lines and insulators often occupy only tiny spatial regions, while the majority of the image is occupied by redundant backgrounds. Traditional global self-attention mechanisms force interactions across all pixel pairs in the entire image; this not only causes the computational complexity to grow quadratically at $\text{O}((\text{HW}{)}^{\text{2}})$ but also introduces a massive amount of noise interference from irrelevant backgrounds, thereby diluting the model’s focus on minute defects. To resolve this issue, this paper adapts the Bi-Level Routing Attention module from BiFormer [44] to serve as the AIBR module. As shown in Fig 6, this module adopts a dynamic sparse strategy of “routing first, computing later,” establishing long-range dependencies only between semantically highly relevant regions by filtering out irrelevant background areas.

Specifically, AIBR first divides the input feature map $\text{X}\in R^{\text{H}\times \text{W}\times \text{C}}$ into S×S non-overlapping physical regions, with each region containing $\frac{\text{HW}}{{\text{S}}^{\text{2}}}$ feature vectors. Query, Key, and Value tensors are generated respectively through linear mapping.

To determine which regions require attention interaction, the model constructs a directed routing graph by calculating the semantic relevance between regions. First, average pooling is performed on the features within each region to obtain region-level query and key vectors. Subsequently, the adjacency matrix between regions is calculated, and a Top-k strategy is utilized to retain the k associated regions with the highest semantic relevance, generating the routing index:


$$\begin{array}{c} {\text{A}}^{\text{r}}={\text{Q}}^{\text{r}}({\text{K}}^{\text{r}}{)}^{\text{T}} \end{array}$$ (4)

$$\begin{array}{c} {\text{L}}^{\text{r}}={\text{Top}}_{\text{k}}({\text{A}}^{\text{r}}) \end{array}$$ (5)

where the i-th row in ${\text{L}}^{\text{r}}$ contains the indices of the k regions that are most semantically relevant to the i-th region, thereby excluding the interference of massive irrelevant background regions.

In the fine-grained computation stage, the model gathers key-value pairs only from the associated regions based on the routing index ${\text{L}}^{\text{r}}$, and calculates the final sparse attention combined with a Local Context Enhancement (LCE) term. Defining Attention(·) as the standard multi-head attention operator, this process can be formally expressed as:


$$\begin{array}{c} \text{O}=\text{Attention}(\text{Q},{\text{K}}^{\text{g}},{\text{V}}^{\text{g}})+\text{LCE}(\text{V})=\text{Softmax}\left(\frac{\text{Q}({\text{K}}^{\text{g}}{)}^{\text{T}}}{\sqrt{{\text{d}}_{\text{k}}}}\right){\text{V}}^{\text{g}}+\text{DWConv}(\text{V}) \end{array}$$ (6)

Through this mechanism, AIBR restricts the attention computation to a small set of key regions filtered by routing. To rigorously illustrate its optimization of complexity, assume the input feature map resolution is H×W and the number of channels is C. The complexity of traditional global self-attention is $\text{4HW}{\text{C}}^{\text{2}}+\text{2}(\text{HW}{)}^{\text{2}}\text{C}$, which grows quadratically with spatial resolution. However, after introducing the S×S division and Top-k sparse routing, the complexity of AIBR becomes:


$$\begin{array}{c} \Omega (\text{AIBR})=\text{4HW}{\text{C}}^{\text{2}}+\text{2}{\text{S}}^{\text{4}}\text{C}+\text{2}\frac{\text{k}}{{\text{S}}^{\text{2}}}(\text{HW}{)}^{\text{2}}\text{C} \end{array}$$ (7)

When the routing parameters are reasonably set (i.e., $\text{k}<{\text{S}}^{\text{2}}$), the dominant quadratic coefficient in the formula is significantly compressed. Combined with the experimental configuration later in this paper (dividing the feature map into ${\text{S}}^{\text{2}}=\text{64}$ sub-regions and retaining k=16 key regions), the sparsity ratio is only 25%. This implies that compared to global self-attention, AIBR substantially reduces the quadratic computational volume. This sparse computation strategy, which compresses the quadratic coefficient, effectively alleviates the computational redundancy introduced by high-resolution feature maps. Simultaneously, this mechanism drives the model to precisely focus on critical defect regions of transmission lines during the feature interaction stage, further enhancing the purity of feature extraction for minute objects and aligning closely with deployment constraints at the edge.

### Efficient group convolution mechanism based on channel shuffle

To address the issues of parameter redundancy and excessive computational overhead in the original RepC3 module of the RT-DETR neck network, this paper introduces a lightweight feature-extraction module, ShuffleC3, inspired by ShuffleNetV2 [45], its design introduces a dual mechanism, “Channel Split” and “Channel Shuffle,” to break the “information silos” of group convolutions while reducing computational complexity by an order of magnitude.

Although traditional group convolution reduces FLOPs, it hinders information flow between groups. Channel shuffle aims to achieve uniform information exchange between groups through dimensional reorganization. Suppose the input feature map X contains C channels, divided into g groups, with each group containing n=C/g channels. The operational flow of channel shuffle S(·) is not a black box; rather, it encompasses two levels: strict dimensional transformation and index remapping.

First, the input dimension (g×n,H,W) is reshaped into (g,n,H,W), then the first two dimensions are swapped (transposed) to (n,g,H,W), and finally flattened back to (g×n,H,W). Let the input channel index be i and the output channel index be j. Channel shuffle essentially executes the following deterministic bijective transformation:


$$\begin{array}{c} \text{j}=(\text{i mod n})\times \text{g}+\left\lfloor \frac{\text{i}}{\text{n}}\right\rfloor \end{array}$$ (8)

where mod represents the modulo operation, and ⌊·⌋ represents the floor operation (rounding down). This formula indicates that the r-th channel of the k-th group in the input feature is precisely mapped to the k-th position of the r-th group in the output feature. Through this “reshape-transpose-flatten” transformation, the model achieves full-channel feature fusion without introducing additional parameters.

**Fig 7:**
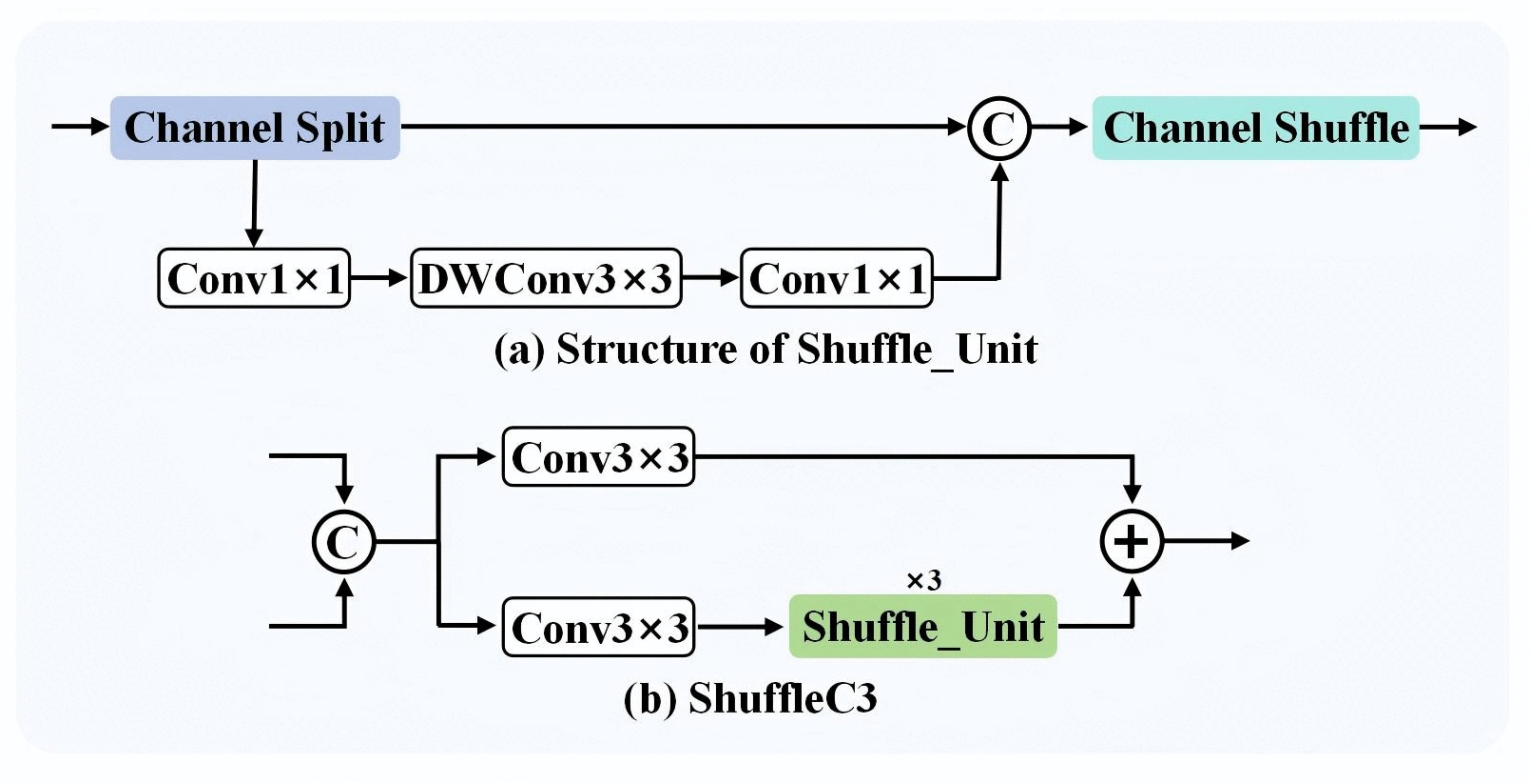
Schematic diagram of the Channel Shuffle principle and structure.

Based on the above principles, the ShuffleC3 module constructs an efficient architecture of “dual-stream residual + cascaded stacking”. First, to reduce the computational overhead, the module uses the Shuffle Unit shown in Fig 7 as its basic unit. This unit utilizes a Channel Split strategy to divide the input feature channels C into two: the first channel branch undergoes identity mapping without any convolution operation, thereby achieving feature reuse and reducing memory access overhead; the second channel branch sequentially executes a 1×1 Conv, a 3×3 DWConv and a 1×1 Conv. After the outputs of the two branches are concatenated along the channel dimension, they are mixed again through the aforementioned channel shuffle operation S(·) to form a complete feature extraction closed loop.

As shown in Fig 7, in the neck design of TL-DETR, the ShuffleC3 module continuously stacks 3 Shuffle Units on the main branch to ensure the extraction capability of deep semantics, and its overall forward propagation process can be expressed as:


$$\begin{array}{c} {\text{Y}}_{\text{out}}=\omega {\left(\text{S}\left(\text{Concat}\left[{\text{X}}_{\text{1}},{\text{F}}_{\text{DW}}({\text{X}}_{\text{2}})\right]\right)\right)}_{\times \text{3}} \end{array}$$ (9)

where ${\text{F}}_{\text{DW}}$ represents the depthwise separable convolution transformation sequence of the second channel branch. This design balances detection accuracy and inference speed while maintaining receptive field and feature-extraction capabilities.

### Cross-spatial learning based on efficient multi-scale attention

At the topmost level of the neck network (S5), after 32 × downsampling, the feature map possesses the richest semantic information but has very low spatial resolution. This low-resolution characteristic makes the spatial structure information of minute defects, such as overhead line wear or insulator flashover, highly susceptible to loss, leading to a phenomenon of “strong semantics but weak localization”. Although the traditional global channel attention mechanism can enhance category features, it further compresses the spatial dimension through global pooling, exacerbating the loss of positional information for minute objects. Therefore, this paper avoids abusing the attention mechanism at all levels; instead, the EMA module [46] is introduced solely at the end of the S5 layer, leveraging its unique Cross-Spatial Learning capability to precisely restore the spatial sensitivity of high-level features.

**Fig 8:**
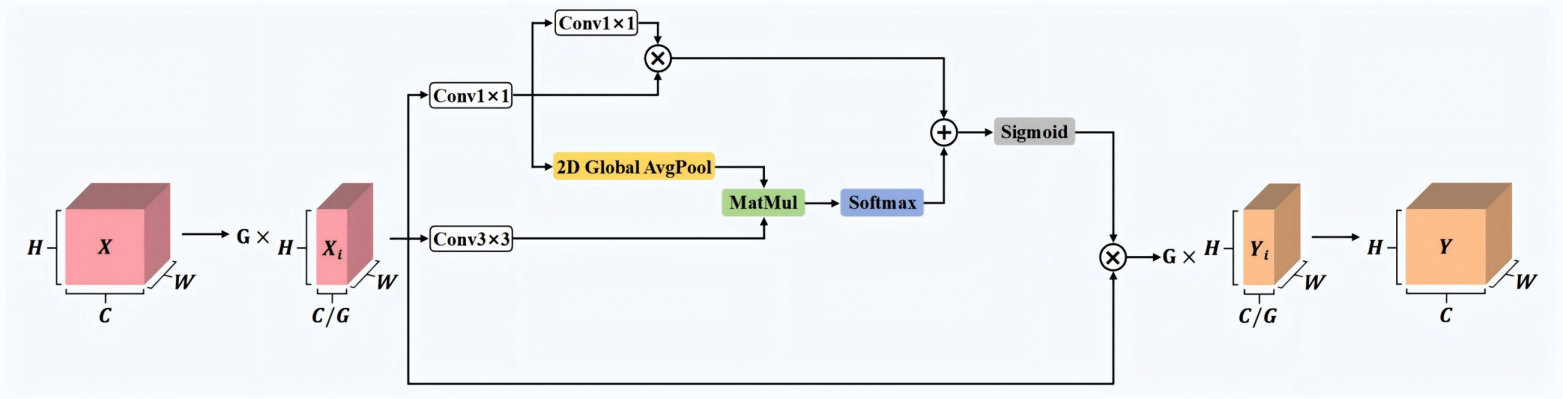
Principle and structure of the EMA module.

As illustrated in Fig 8, the EMA module discards traditional dimensionality reduction operations. It first groups the input feature X along the channel dimension and utilizes two 1×1 Conv branches and one 3×3 Conv branch to extract contextual features of different scales in parallel. Subsequently, to overcome the spatial information loss caused by global average pooling, The prior EMA framework utilizes a bidirectional cross-spatial interaction strategy. It performs 2D global average pooling on the output of the 1×1 Conv branch, compressing the spatial dimension information into a global descriptor, thereby capturing the long-range dependencies of the image. Then, the encoded global descriptor undergoes a matrix Dot Product operation with the local feature map of the 3×3 Conv branch. This operation is no longer a simple channel weighting but allows the global context to directly “modulate” the local pixel features.

Let the output of the 1×1 Conv branch be ${\text{F}}_{\text{1}\times \text{1}}$, the output of the 3×3 Conv branch be ${\text{F}}_{\text{3}\times \text{3}}$, and the 2D global average pooling be ${\text{AvgPool}}_{\text{2d}}$. The attention map M generated by cross-spatial learning can be formally expressed as the interaction result normalized by Softmax:


$$\begin{array}{c} \text{M}=\text{Softmax}\left(\frac{{\text{AvgPool}}_{\text{2d}}({\text{F}}_{\text{1}\times \text{1}})\cdot \overset{\text{T}}{\underset{\text{3}\times \text{3}}{\text{F}}}}{\sqrt{\text{d}}}\right) \end{array}$$ (10)

where d is the scaling factor. This formula indicates that EMA establishes a dual relationship between “global channel statistics” and “local spatial features”, enabling the model to utilize full-image contextual information to calibrate the feature response of every minute local area.

Finally, the module aggregates the attention weights generated by the two parallel branches after cross-spatial interaction, generates the final pixel-level weight map through the Sigmoid function, and recalibrates the original input feature X:


$$\begin{array}{c} {\text{Y}}_{\text{out}}=\text{X}\cdot \text{Sigmoid}(\text{Aggregation}({\text{M}}_{\text{1}\to \text{3}},{\text{M}}_{\text{3}\to \text{1}})) \end{array}$$ (11)

Through this mechanism, the EMA module not only preserves the crucial spatial information of minute defects but also effectively suppresses background noise by aggregating global context, thereby improving the localization precision of TL-DETR for fine-grained defects on transmission lines.

## Results

### Experimental details

#### Transmission line defect detection datasets.

Given that existing research on transmission line defect detection is mostly confined to binary classification (i.e., “presence or absence of defects”), and that different types of subtle defects correspond to vastly different disposal strategies in actual operation and maintenance, fine-grained classification has greater practical engineering value. To this end, this study selects the CableInspect-AD expert-annotated dataset, jointly released by Hydro-Québec and the Mila – Quebec Artificial Intelligence Institute, as the research foundation [47]. This original open dataset contains 4,798 high-resolution images, including 2,639 positive samples with abnormal defects and 2,159 negative samples without defects, primarily covering typical line-defect data under monochromatic backgrounds or in controlled laboratory environments.

**Fig 9:**
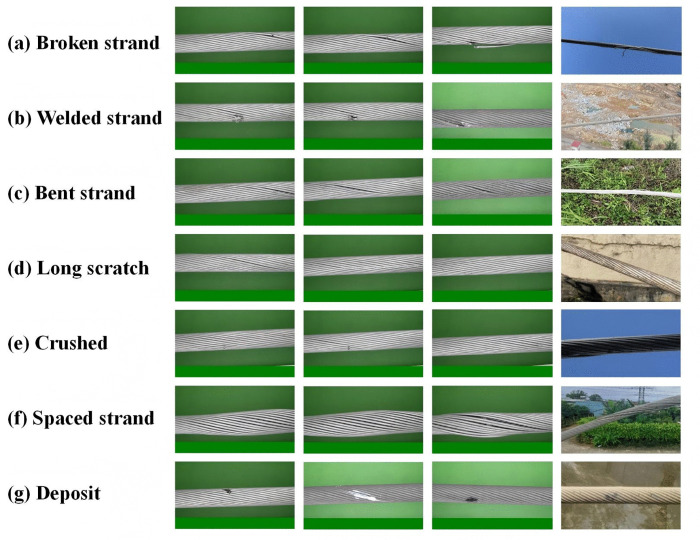
Representative samples of various overhead line defect categories in the constructed CableInspect-ADs dataset under complex natural backgrounds.

Given that controlled environments struggle to fully capture the complex and variable real-world inspection conditions, this study deliberately introduced real engineering scene samples to enhance model robustness, constructing the CableInspect-ADs extended dataset comprising 5,000 images, as shown in Fig 9. Specifically, we added 202 engineering images involving actual power line field collection from the open industrial dataset MulticableData to the original dataset, while conducting strict screening to ensure no overlap between the newly added samples and the original dataset.

Although the newly added real-world inspection images constitute only a small portion of the extended dataset, acquiring images of subtle defects (such as broken or spaced strands) under complex natural backgrounds in the field of power inspection is extremely costly. Therefore, rather than blindly pursuing data volume, this study positions these 202 images as high-value “Hard Examples.” By introducing interference such as complex backgrounds, the design aims to break the ideal distribution assumption of original laboratory data and simulate the inherent “long-tail distribution” and “small-sample” pain points in industrial applications. This design is intended to rigorously evaluate the model’s detection performance in complex real-world backgrounds and verify whether the multi-scale feature enhancement and dynamic sparse attention architecture can effectively extract defect features. As discussed in the discussion and conclusions section, exploring and solving this typical “data scarcity” dilemma is a core motivation and an important future research direction for this study.

Simultaneously, for the detection task involving key transmission line fittings, we also collected a large dataset to construct the Transmission-Line-Fittings dataset. This dataset contains three sub-datasets: an insulator defect dataset with 3,000 images (defect types include Flashover, Lose, and Damaged); a tower bolt defect dataset with 857 images (defect types include Lose and Side); and a vibration damper defect dataset with 1,000 images (the defect type is Defect). This dataset provides data support for verifying the strong generalization capabilities of our subsequent model.

The experiments in this paper primarily use the CableInspect-ADs dataset, while the Transmission-Line-Fittings dataset serves as the generalization experiment, providing data to demonstrate that the TL-DETR model still achieves excellent results in detecting defects on important transmission line fittings. All datasets were randomly split into a training set (70%), a validation set (20%), and a test set.

### Training settings

The experiments in this study were conducted on Windows 11, with the hardware configuration including an Intel(R) Xeon(R) Platinum 8352V CPU (@ 2.10GHz) and an NVIDIA RTX 4090 GPU (24GB VRAM). Detailed environmental parameters are shown in Table 1. To ensure reproducibility and fairness, this study set the global random seed to 0 and ran a single full training cycle with the deterministic algorithm mode enabled. Regarding the fine-tuning strategy, TL-DETR and all comparison models uniformly loaded the official COCO 2017 pre-trained weights for transfer learning. This ensures that all algorithms share the same optimization starting point, thereby objectively reflecting the impact of architectural improvements on performance.

**Table 1:** Experimental environment configuration.

Parameters	Settings
**Operating systems**	Windows 11
**GPU**	NVIDIA RTX 4090
**Deep learning framework**	Pytorch 2.3.0
**Accelerated computing framework**	CUDA 12.4
**Programming Languages**	Python 3.12.3

Model training uniformly adopted Automatic Mixed Precision (AMP, FP16) to optimize computational efficiency, with 200 training epochs and a batch size of 16. Targeting the characteristics of subtle defects in transmission lines, this study formulated a specific data augmentation strategy: Mosaic data augmentation was enabled with a probability of 1.0 to enrich object scales and background complexity, and random horizontal flipping (probability of 0.5) along with HSV color jittering (hue h = 0.015, saturation s = 0.7, value v = 0.4) were employed to enhance the model’s robustness against complex lighting and viewing angles. Considering that Mixup augmentation and Perspective transformation might cause blurring or excessive deformation of fine-grained object features, this study deliberately disabled them during the experiments. Before being fed into the network, all input images were adaptively resized to 640 × 640 using the Letterbox strategy. Specific training parameter settings are detailed in Table 2.

**Table 2:** Experimental parameter configuration.

Parameters	Values
**Image size**	640 × 640
**Epochs**	200
**Batch size**	16
**Optimizer**	AdamW
**Learning rate**	0.0001
**Decay**	0.0005

### Evaluation metrics

To evaluate the proposed method, precision, recall, average precision (AP), and inference speed are adopted as performance metrics. Precision measures the proportion of correctly classified positive samples among all detected positive samples, while recall represents the proportion of detected positive samples among all actual positive samples. Their definitions are as follows:

Precision:


$$\begin{array}{c} \text{P}=\frac{\text{TP}}{\text{TP}+\text{FP}} \end{array}$$ (12)

Recall:


$$\begin{array}{c} \text{R}=\frac{\text{TP}}{\text{TP}+\text{FN}} \end{array}$$ (13)

TP (True Positive) refers to samples predicted as positive that are actually positive; FP (False Positive) refers to samples predicted as positive that are actually negative; FN (False Negative) refers to samples predicted as negative that are actually positive.

The Average Precision (AP) for a single category label is defined as shown in the formula:


$$\begin{array}{c} \text{AP}=\int_{\text{0}}^{\text{1}}\text{P}(\text{R})\text{dR} \end{array}$$ (14)

The mean Average Precision (mAP) represents the ratio of the sum of the average precisions of all labels to the total number of categories M. The definition of mAP is shown in the formula:


$$\begin{array}{c} \text{mAP}=\frac{\sum_{\text{m}=\text{1}}^{\text{M}}\text{AP}}{\text{M}} \end{array}$$ (15)

The performance of recall and precision is equally crucial. Therefore, this study also adopts the F1 score as an additional performance metric. The F1 score represents the harmonic mean of precision and recall, effectively balancing the two metrics. The F1 score is expressed by the formula:


$$\begin{array}{c} \text{F1}=\text{2}\cdot \frac{\text{P}\cdot \text{R}}{\text{P}+\text{R}} \end{array}$$ (16)

### Experimental analysis

#### Ablation study of the TL-DETR model.

**Fig 10:**
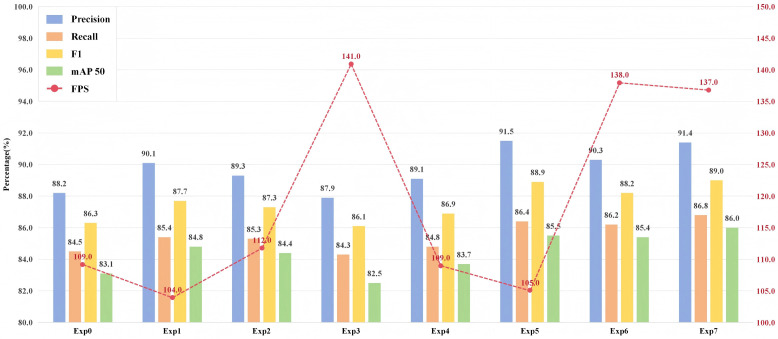
Comparison results of the ablation study.

In this study, we randomly selected 500 images from the CableInspect-ADs dataset as the test set for the entire experiment. Each module discussed in the methods section was sequentially added to the RT-DETR model to verify the effectiveness of the proposed modules and their impact on model performance. Table 3 and Fig 10 present the comprehensive test results of each module, where A, B, C, and D represent the ResNet-50-TL backbone network introducing the MSFE module, the AIBR module, the ShuffleC3 module, and the EMA module, respectively.

**Table 3:** Results of the ablation study for each module on the CableInspect-ADs dataset.

Name	Model	P	R	F1	mAP50	mAP50-95	Param/10^6^	GFLOPS	FPS
**Exp0**	RT-DETR	0.882	0.845	0.863	0.831	0.504	42.8	130.5	109
**Exp1**	+A	0.901	0.854	0.877	0.848	0.515	43.7	132.8	104
**Exp2**	+B	0.893	0.853	0.873	0.844	0.511	42.3	130.3	112
**Exp3**	+C	0.879	0.843	0.861	0.825	0.504	35.3	93.2	141
**Exp4**	+D	0.891	0.848	0.869	0.837	0.508	42.8	130.5	109
**Exp5**	+A + B	0.915	0.864	0.889	0.855	0.521	43.0	132.6	105
**Exp6**	+A + B + C	0.903	0.862	0.882	0.854	0.519	35.5	95.3	138
**Exp7**	+A + B + C + D	0.914	0.868	0.890	0.860	0.522	35.5	95.3	137

After introducing the MSFE module based on the baseline experiment (Exp0), both the Precision and mAP50 of the model improved significantly, increasing from 88.2% to 90.1% and from 83.1% to 84.8%, respectively. This proves that the multi-scale parallel pathway is highly effective in enhancing fine-grained feature capture. Meanwhile, thanks to the introduction of depthwise separable convolutions, the model incurs minimal computational overhead while obtaining a large receptive field. Subsequently, the introduction of the AIBR module (Exp2) increased the mAP50 by 1.3% while elevating the FPS to 112, verifying the high efficiency of the dynamic sparse attention mechanism in filtering background noise. After further introduction of the ShuffleC3 module (Exp3), the model achieved an inference speed of 141 FPS, significantly reducing computational redundancy. However, it also experienced a slight decrease in Precision and mAP50, reflecting the natural trade-off between lightweight design and feature expression. In actual UAV edge inspection scenarios, constrained by the power consumption and computing power of airborne equipment, sacrificing a small amount of theoretical accuracy in exchange for a substantial increase in inference speed can effectively prevent frame drops and processing lag during dynamic flight; such a trade-off is reasonable and necessary in engineering applications.

In the module combination experiments, the integration of MSFE, AIBR, and ShuffleC3 (Exp6) achieved balanced performance of 90.3% Precision and 138 FPS, demonstrating that a lightweight design can effectively compensate for the computational overhead introduced by feature enhancement modules. Meanwhile, the final fully integrated model (Exp7) achieved the best comprehensive performance, with its Precision, Recall, F1 score, and mAP50 reaching 91.4%, 86.8%, 89.0%, and 86.0%, respectively. This configuration mitigates speed loss through efficient architectural design while maintaining the advantages of multi-scale fusion and global recalibration. Ultimately, compared with the baseline model, the proposed TL-DETR model improved Precision, Recall, F1 score, and mAP50 by 3.2%, 2.3%, 2.7%, and 2.9%, respectively, and its inference speed increased to 137 FPS.

It is worth mentioning that the model’s parameter count (35.5 M) and floating-point operations (95.3 GFLOPS) are also optimized compared to the baseline model (42.8 M, 130.5 GFLOPS). Although the RT-DETR model has been proven to possess real-time detection capabilities and meet general deployment requirements [17], TL-DETR demonstrates superior edge adaptability by further compressing computational redundancy. Preliminary theoretical analysis indicates that on mainstream embedded devices (such as the NVIDIA Jetson Orin NX, which provides 100 TOPS of AI computing power), the computational demand of this model is well below the device’s upper limit for computing power. This implies that, while maintaining high detection performance, the improved model has the hardware foundation to reserve computing resources for concurrent tasks, such as UAV mission payloads and flight control, thereby preliminarily achieving a balance among precision, speed, and computational overhead.

### Internal ablation study of the MSFE module

To further verify the synergistic effectiveness of the multi-scale perception branches within the MSFE module, this study designed an ablation experiment targeting the module’s internal structure. The compared versions include: a sub-version retaining only the local branch (a parallel combination of 3×3 and 5×5 DWConv), a sub-version retaining only the global branch (7×7 DWConv), and the complete MSFE module. Regarding the selection of performance evaluation metrics, considering that the safety hazards caused by “missed detections” in actual transmission line inspections are far greater than those caused by “false alarms,” this internal ablation study specifically selects Recall, R(%), as the core constraint metric to examine the capture capability of different branches for weak defects, supplemented by mAP50(%) to evaluate its comprehensive detection accuracy.

The experimental results are shown in Table 4. The experimental results indicate that the local branch performs better at capturing the minute edges and geometric textures of defect targets, thereby improving the model’s positioning accuracy for fine-grained objects; while the global branch enhances the model’s ability to capture contextual information under complex backgrounds by expanding the effective receptive field. Compared to a single perception path, the complete MSFE module with a multi-scale parallel topology achieves significant improvements across all detection metrics by deeply fusing local fine-grained features with global background information. This objectively verifies the effectiveness of the “split-transform-merge” strategy in enhancing the feature expression of fine-grained transmission line defects, demonstrating that perception branches of different scales exhibit significant synergistic and complementary effects in small-object detection tasks.

**Table 4:** Internal Ablation Study Results of Different Branches in the MSFE Module.

Name	Model Structure	R(%)	mAP50(%)
**MSFE-Local**	Only Local Branch (3×3+5×5)	85.8	85.2
**MSFE-Global**	Only Global Branch (7×7)	84.3	83.8
**MSFE-Full**	Parallel Topology (Local + Global)	86.8	86.0

### Hyperparameter sensitivity analysis of the AIBR module

To verify the rationality of the key hyperparameter values and ensure their robustness under complex transmission-line backgrounds, this paper conducted a sensitivity analysis of the routing parameters (the number of region partitions S and the sparsity degree k) of the AIBR module. All experiments were conducted on the CableInspect-ADs dataset, and the baseline configuration was consistent with Exp7 mentioned above.

First, this paper fixed the region partition parameter at S=8 (i.e., dividing the feature map into 64 physical sub-regions) and explored the optimal feature sparsity ratio by gradually adjusting the number of routing k. The experimental results are shown in Table 5. A clear performance trade-off can be observed from the table: when k is small (e.g., k=4 or k=8), due to the excessively high sparsity, the model filters out too much information and loses the key context necessary for capturing long-range dependencies, resulting in varying degrees of decline in both Recall and mAP50. Conversely, when k increases to 64 (degenerating into global attention), although the receptive field achieves full coverage, a large amount of useless background noise (such as large areas of vegetation, sky, etc., in the background) is simultaneously introduced, which paradoxically dilutes the feature representation of weak defects. Experiments demonstrate that when k=16 (i.e., the sparsity ratio is 25.0%), the model achieves a balance between effectively filtering background noise and retaining key semantics.

**Table 5:** Sensitivity analysis of the routing parameter 𝐤 in the AIBR module.

k	k/S^2^	R(%)	mAP50(%)
**4**	6.3%	84.0	83.3
**8**	12.5%	85.7	85.2
**16**	25.0%	86.8	86.0
**32**	50.0%	86.0	85.4
**64（Global）**	100.0%	85.5	85.2

Under the premise of determining 25.0% as the optimal sparsity ratio, to further explore the impact of spatial partition granularity S on detection performance and inference speed, this paper compared the performances when S takes the values of 4, 8, and 16, respectively (synchronously adjusting k to maintain the 25% sparsity ratio). The results are shown in Table 6. The experiment found that when S=4, because the receptive field of a single routing region is too large, the model struggles to precisely localize fine-grained defects. When S=16, although the partition is finer, the sharp increase in the number of generated sub-regions significantly increases the overhead of dynamic routing calculation, resulting in a corresponding decline in FPS.

**Table 6:** Impact Analysis of Different Spatial Partition Granularities 𝐒.

S	k	R(%)	mAP50(%)	FPS
4	4	84.5	83.9	137
8	16	86.8	86.0	137
16	64	86.4	85.7	132

Considering the trade-off between speed and precision in edge deployment, S=8 is a better choice for the current input resolution (640×640). This setting is highly correlated with the resolution; at the current resolution, the sub-region granularity generated by S=8 can effectively cover the typical geometric dimensions of transmission line defects, and can eliminate redundant noise at an appropriate scale through the AIBR mechanism, thereby achieving better perceptual efficacy.

### Configuration and deployment position analysis of the EMA module

To verify the rationale for deploying the EMA module at the end of the S5 layer, this study investigated the performance of the attention mechanism at different feature levels (S3, S4, S5) and their combination (S3 + S4 + S5), aiming to explore the optimal balance between perceptual gain and computational overhead. The experimental results are shown in Table 7.

**Table 7:** Impact Analysis of EMA Deployment Positions.

EMA Placement	R(%)	mAP50(%)	FPS
**S3**	85.7	85.2	132
**S4**	86.0	85.5	134
**S5(Ours)**	86.8	86.0	137
**S3 + S4 + S5.**	87.0	86.1	128

Differences in the attention mechanism’s sensitivity to features at different levels can be observed in the experimental data. When the EMA is deployed at the S3 or S4 layers—which have higher spatial resolution but relatively shallower semantics—the introduction of attention yields a marginal improvement in recall but contributes little to overall mAP50, as shallow features contain substantial high-frequency background noise. Simultaneously, because high-resolution feature maps increase the volume of tensor operations, the inference speed (FPS) slightly drops to 132 and 134, respectively. In contrast, applying the EMA to the S5 layer, which is rich in semantic information, helps alleviate localization errors for subtle defects during continuous downsampling, yielding greater accuracy while maintaining a real-time inference speed of 137 FPS. Furthermore, when the EMA is introduced simultaneously across all levels (S3 + S4 + S5), although the mAP50 reaches 86.1%, the FPS decreases due to the additional memory access overhead introduced by module stacking. Therefore, concentrating EMA deployment in deep networks is an effective approach to balance detection accuracy and inference efficiency.

Regarding the hyperparameter configuration within the module, the number of channel groups g (i.e., feature grouping granularity) is critical in determining the capability of parallel feature representation. In the TL-DETR implementation of this study, which comprehensively considers feature dimensions and hardware computing constraints, this parameter g is set to 32. The logic behind this setting is as follows: dividing deep features into 32 parallel sub-groups along the channel dimension enables the model to capture richer multi-scale spatial correlations without significantly increasing the parameter count. If the number of groups is too small, it restricts the parallel representation capability of multi-dimensional features, leading to insufficient feature utilization; if the grouping is too fragmented, it disrupts the semantic coherence of feature channels and reduces the computational continuity of the GPU by increasing the frequency of memory accesses. Experimental validation demonstrates that g=32 achieves the optimal trade-off between the richness of defect feature extraction and the inference efficiency of edge devices.

### Comparative experiments

To verify the effectiveness of the proposed model, we conducted in-depth comparative experiments between TL-DETR and various current mainstream detection algorithms on both the extended CableInspect-ADs dataset and the original public CableInspect-AD dataset. First, the comparison results on the CableInspect-ADs dataset are presented in Table 8. To ensure absolute fairness in the comparison and to rule out interference caused by random initialization, all compared models uniformly utilized the official pre-trained weights from the COCO 2017 dataset for initialization. Regarding the evaluation of inference speed (FPS), to objectively reflect the differences in computational efficiency inherent to the model architectures themselves and to eliminate the interference of acceleration libraries, the speed measurements for all experiments in this study (including the aforementioned ablation studies and the comparative experiments in the comparative experiments section) were conducted under unified benchmark conditions. Specifically, pure PyTorch forward inference tests were performed using half-precision floating-point format (FP16) on a single NVIDIA RTX 4090 GPU, with a fixed batch size of 16, and without introducing any hardware-specific inference acceleration engines such as TensorRT or ONNX Runtime. This configuration ensures that the reported FPS metrics can serve as an objective, algorithm-level benchmark reference independent of underlying deployment optimizations.

**Table 8:** Detection performance of various models on the CableInspect-ADs dataset.

Model	P	R	F1	mAP50	mAP50-95	Param/10^6^	GFLOPS	FPS
**YOLOv5-l** [48]	0.870	0.835	0.852	0.801	0.481	46.5	109.3	155
**YOLOv8-l** [49]	0.873	0.832	0.852	0.803	0.484	43.7	160.1	135
**YOLOv11-l** [50]	0.872	0.830	0.850	0.808	0.489	25.3	86.9	161
**YOLOv12-l** [51]	0.861	0.838	0.849	0.784	0.473	28.4	91.5	138
**YOLOv13-l** [52]	0.879	0.842	0.860	0.822	0.497	29.6	90.8	140
**RT-DETR** [17]	0.882	0.845	0.863	0.831	0.504	42.8	130.5	109
**TL-DETR**	0.914	0.868	0.890	0.860	0.522	35.5	95.3	137

Regarding the selection logic of comparison models, this study covers several milestone architectures in the field of object detection, including the most widely used and community-optimized YOLOv5 and YOLOv8 baseline models; the official latest version, YOLOv11; and the recently released YOLOv12 and YOLOv13 models, which improve upon the balance between precision and speed. To ensure fairness and rigor in the comparison, all YOLO models use the L (Large) version, which achieves the highest detection level of the baseline models through a deeper network architecture, thereby verifying the performance advantages of the TL-DETR model under the strictest accuracy benchmarks.

From the perspective of model architecture evolution, the latest single-stage convolutional neural networks, YOLOv11-l and YOLOv13-l, achieved strong performance on the CableInspect-ADs dataset, with mAP50 exceeding 80%. This represents the state of the art in convolutional neural networks (CNNs) for feature fusion and computational resource allocation. However, the TL-DETR proposed in this paper, leveraging Transformers’ long-range modeling capabilities, further increased Precision to 91.4%, significantly outperforming the highest level of the current YOLO series. This shows that, for inspection tasks involving subtle defects such as those in transmission lines, the end-to-end Transformer architecture has greater potential to capture fine textures than traditional convolutional networks, enabling more effective extraction of key defect features.

Regarding the detection accuracy of tiny objects on transmission lines, TL-DETR demonstrates a significant advantage. Experimental results show that the mAP50 of TL-DETR reached 86.0%, an improvement of 2.9 percentage points over the baseline RT-DETR model. This improvement in accuracy does not rely on blindly stacking parameters but originates from the preservation of geometric details at the source by the MSFE backbone network and the restoration of spatial sensitivity in high-level features by the EMA module. Compared with the latest YOLOv13-l, TL-DETR leads by 3.5% in Precision and is 2.6% higher in Recall, indicating that the model effectively addresses the challenge of fine-grained defects on overhead lines being easily submerged in deep networks, effectively reducing the missed-detection rate in industrial inspections.

In terms of the dynamic balance between real-time performance and computational overhead, TL-DETR also performs excellently. Benefiting from the elimination of computational redundancy by the ShuffleC3 module and the dynamic sparse strategy of the AIBR module, the model achieved 137 FPS. Although this performance is slightly inferior to that of some YOLO models, it still meets the deployment requirements for edge testing.

Furthermore, to exclude the performance contingency brought by a single random initialization, this study conducted independent repeated experiments on the baseline RT-DETR and the proposed TL-DETR using three other different global random seeds (Seed = 10, 100, 1000). Specifically, the three mAP50 results of the baseline RT-DETR were 82.0%, 83.2%, and 83.3% (mean: 82.8% ± 0.72%), while the three results for TL-DETR were 85.9%, 86.0%, and 86.2% (mean: 86.0% ± 0.15%). Statistical analysis shows that TL-DETR outperforms the baseline model by a significant margin (independent-samples t-test, p < 0.05). This statistical result also shows that the effectiveness of the architectural improvements proposed in this paper is robust and does not stem from random initialization bias in the weights.

Simultaneously, to verify the actual effect of the proposed architectural optimization and reduce the interference of dataset expansion on performance evaluation, we conducted control experiments on the original public dataset CableInspect-AD, with the results shown in Table 9. The experimental results show that under the original working conditions, without any newly added samples, the mAP50 of TL-DETR still improved over the baseline RT-DETR model. This further explains that the improvement in detection performance stems from the optimization of structures such as the MSFE backbone network and the AIBR introduced in this paper, rather than from purely expanding dataset scale or changing background complexity. This provides objective validation of the effectiveness of the proposed improvement strategy for fine-grained detection tasks.

**Table 9:** Detection performance of various models on the CableInspect-AD dataset.

Model	P	R	F1	mAP50	mAP50-95	Param/10^6^	GFLOPS	FPS
**YOLOv5-l**	0.885	0.848	0.866	0.816	0.495	46.5	109.3	155
**YOLOv8-l**	0.887	0.845	0.866	0.819	0.500	43.7	160.1	135
**YOLOv11-l**	0.888	0.842	0.864	0.825	0.503	25.3	86.9	161
**YOLOv12-l**	0.876	0.852	0.864	0.802	0.487	28.4	91.5	138
**YOLOv13-l**	0.893	0.855	0.874	0.840	0.510	29.6	90.8	140
**RT-DETR**	0.896	0.858	0.877	0.846	0.518	42.8	130.5	109
**TL-DETR**	0.926	0.881	0.903	0.878	0.535	35.5	95.3	137

**Fig 11:**
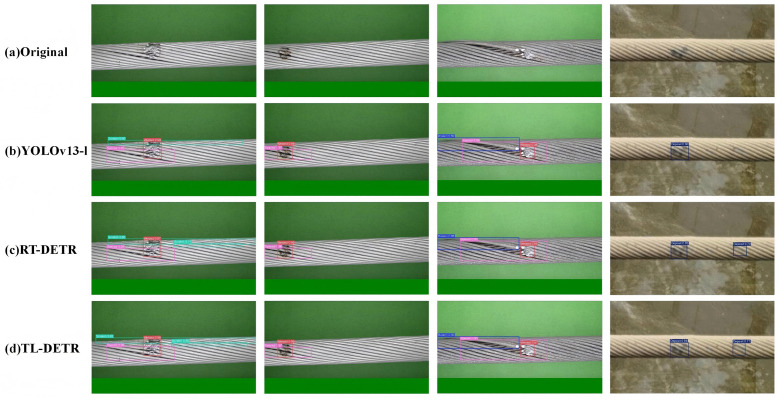
Visualized results of different detection models on the CableInspect-ADs dataset.

In addition to quantitative metrics, qualitative visual evaluation better reflects the model’s robustness on samples of varying difficulty. Fig 11 compares the detection instances of various models on the CableInspect-ADs dataset. The experimental results show that although existing state-of-the-art (SOTA) models (YOLOv13-l and RT-DETR) possess strong general detection capabilities, they often miss subtle defects, such as broken strands and wear on overhead lines, due to insufficient feature extraction. Conversely, the TL-DETR model, with its network architecture optimized for minute objects, exhibits better perception performance. This model not only can isolate weak defect features from complex backgrounds but also effectively curbs false alarms and missed detections, reflecting higher detection reliability.

**Fig 12:**
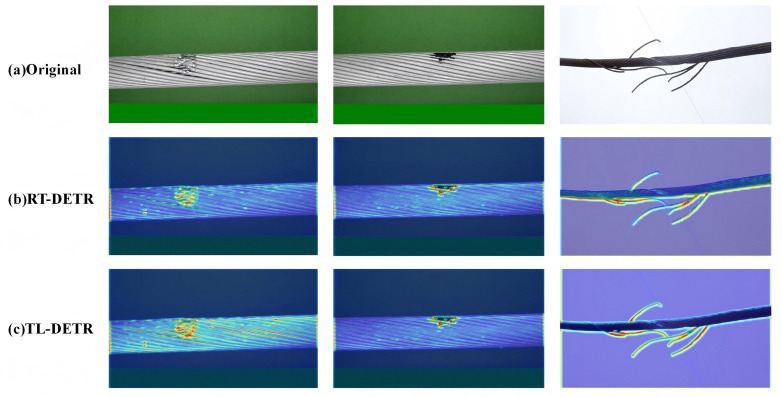
GradCAM++ heatmap comparison between RT-DETR and TL-DETR on representative defect samples.

Meanwhile, to further explore the model’s internal decision-making process and verify its interpretability, this paper uses the GradCAM++ technique [53] to generate class activation heatmaps (as shown in Fig 12). The color depths in the heatmaps map the feature extraction network’s response intensity across different regions of the image, with the red-highlighted areas representing the model’s core focus points. Through comparative analysis, it can be found that the attention distribution of the baseline model, RT-DETR, is relatively divergent and susceptible to background texture interference; whereas the TL-DETR model shows better focusing, with high-response regions corresponding to the actual defect locations. This significant attention calibration effect highlights the improvement in suppressing background noise and locking onto key defect features provided by the proposed model.

### Generalization experiments

To further verify the generalization ability and robustness of the proposed TL-DETR model in multi-scenario and multi-scale target tasks, this paper conducts supplementary generalization experiments on three key components: insulators, bolts, and vibration dampers, using the Transmission-Line-Fittings dataset. Unlike the surface defect detection task of overhead lines, this fittings dataset exhibits a significant span in target scales. On the one hand, vibration dampers and conventional bolts account for a relatively large proportion of the collected images, and their visual features are relatively salient. On the other hand, local damage to fittings, especially insulator flashovers and minor breakages, often occurs in areas with severe background interference, and the defect areas occupy only a few pixels in the entire high-resolution inspection image. Small-object detection under complex backgrounds imposes stringent requirements on the model’s fine-grained feature perception and background noise filtering capabilities, which is exactly the core motivation behind constructing the TL-DETR architecture in this paper.

**Fig 13:**
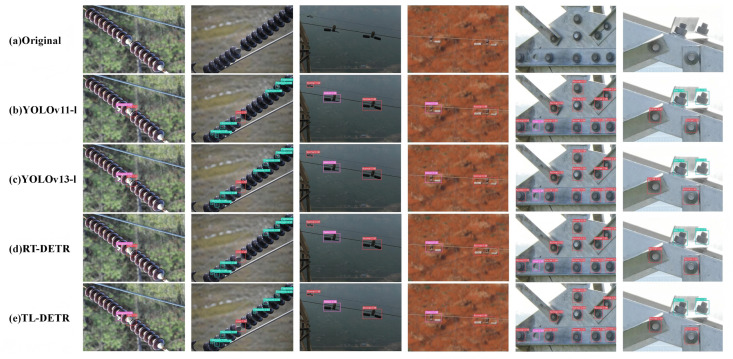
Visualized results of different detection models on the Transmission-Line-Fittings dataset.

Table 10 and Fig 13 present the generalization performance results of various models on the Transmission-Line-Fittings dataset across different transmission line fittings, revealing a mapping relationship between model architectural features and target scales. In fine-grained detection tasks, such as insulator detection, characterized by severe background interference and small defect areas, the proposed TL-DETR model achieves the best performance. With Precision and mAP50 reaching 0.897 and 0.827, respectively, it comprehensively surpasses YOLOv11-l, YOLOv13-l, and the baseline RT-DETR model. This effectively validates the advantages of TL-DETR in filtering redundant backgrounds and accurately capturing minute defect features. Conversely, when faced with conventional fittings with larger scales and more prominent visual contours, such as vibration dampers and bolts, YOLOv11-l exhibits superior representation extraction, thanks to its deep convolutional architecture and optimized spatial feature fusion network, achieving peak mAP50 scores of 0.958 and 0.938, respectively. Although the peak metrics of TL-DETR on these large-scale targets are slightly lower than those of YOLOv11-l—which is aggressively optimized for general large-object detection—its performance on vibration dampers and bolts remains robust and significantly outperforms the baseline RT-DETR and the latest YOLOv13-l. In summary, the generalization experiments objectively demonstrate that TL-DETR has achieved its core objective of substantially improving the perception of small objects in complex backgrounds while maintaining strong generalization resilience for conventional multi-scale targets, thereby meeting the practical engineering requirements for comprehensive multi-component inspections of transmission lines.

**Table 10:** Detection performance of various models on the Transmission-Line-Fittings dataset.

Model	Insulator			Screw			Damper		
	P	mAP50	mAP50-95	P	mAP50	mAP50-95	P	mAP50	mAP50-95
**YOLOv11-l**	0.851	0.809	0.495	0.932	0.938	0.632	0.956	0.958	0.748
**YOLOv13-l**	0.875	0.813	0.505	0.880	0.875	0.588	0.893	0.832	0.582
**RT-DETR**	0.882	0.819	0.503	0.897	0.885	0.594	0.911	0.894	0.697
**TL-DETR**	0.897	0.827	0.511	0.912	0.896	0.603	0.927	0.914	0.711

## Discussion and conclusions

Addressing the challenges posed by transmission line defects—fine-grained, low-contrast, and highly susceptible to background interference—this paper proposes TL-DETR, a high-precision defect-detection model designed for edge deployment. In terms of architectural design, this study constructs the ResNet-50-TL backbone network incorporating the MSFE module. By synergistically integrating multi-scale pathways and depthwise separable convolutions, it effectively expands the receptive field with minimal computational overhead, thereby preserving the weak geometric details of defects at the source. Supplemented by the dynamic sparse mechanism and lightweight design of the neck network, the model achieves precise focusing on key defect regions within complex power grid backgrounds.

Experimental results demonstrate that TL-DETR achieves competitive detection performance on the CableInspect-ADs dataset, with a precision of 91.4% and mAP50 of 86.0%, representing improvements of 3.2% and 2.9% over the baseline RT-DETR model, respectively. This demonstrates that the proposed architecture can effectively alleviate the problem of subtle defect features being submerged in deep networks. Meanwhile, generalization experiments on the fitting dataset confirm that the model maintains strong reliability across targets of varying sizes, including insulators, vibration dampers, and bolts.

Despite achieving notable improvements in performance, this study still has objective limitations. At the hardware level, the current inference speed evaluation is primarily based on a high-end desktop graphics card (NVIDIA RTX 4090). Constrained by differences in underlying scheduling mechanisms, the current FPS metric serves more as a theoretical benchmark for the effectiveness of the architectural optimization. In terms of quantitative metrics, TL-DETR has 35.5M parameters and a computational complexity of 95.3 GFLOPs; although it possesses the hardware foundation for smooth execution on edge computing platforms, end-to-end on-device verification on actual embedded equipment is still required. Furthermore, at the data level, certain critical defects in power inspections occur infrequently and incur high annotation costs. This results in generalization challenges for the model when confronted with novel defect categories characterized by extremely scarce “small samples.”

Future research will focus on the following directions. First, converting TL-DETR into a TensorRT engine to conduct real-world on-device performance verification and low-precision quantization deployment on actual UAV-borne computing platforms. Second, exploring Small-sample Learning strategies to further enhance the model’s representation capability for scarce defects under limited samples, thereby strengthening the algorithm’s engineering practicability in the actual operation and maintenance of power grids.
